# The big problem of the missing cytology slides

**DOI:** 10.1186/1742-6413-1-3

**Published:** 2004-09-24

**Authors:** Ken M Gatter

**Affiliations:** 1 Department of Pathology, L471, Oregon Health and Sciences University, Portland, Oregon 97239, USA

**Keywords:** cytology, Pap, Pap smears, litigation, law

## Abstract

Cytology slides are often unique and irreplaceable. Unlike surgical pathology cases, where additional paraffin sections can be cut, cytology slides often cannot be duplicated because there are only a few direct smears or the diagnostic material is present on a single slide. Cytology slides are often "sent out" to other physicians, laboratories or hospitals, typically so that the pathologist at the institution where the patient will receive treatment can review the slides. Less often, a cytology lab sends out the slides for a second opinion or as part of the discovery process in a lawsuit, where they may or may not be defendants. Rarely, unique and irreplaceable cytology slides are lost. This article presents a hypothetical scenario that is based on reported state appellate court decisions. The article discusses some of the legal issues that will affect the defendant cytologist/cytology lab and the "expert cytologist," and suggests some steps a cytologist/cytology lab can take to minimize the risk of repercussions from a lost unique and irreplaceable cytology slide.

## 1. What is already known on this topic?

A WestLaw search (similar to PubMed, but searches state and federal cases and statutes as well as commentary) uncovered only a handful of reported appellate cases that directly applied to the issue of lost cytology slides. There are many cases and statutes dealing with lost and altered evidence (other than cytology slides), but the circumstances surrounding cytology slides are unique. I did not see any previous reviews or commentary specifically addressing the topic. Notwithstanding the relative paucity of on-point legal authority, the issue is one commonly addressed by cytology laboratories and cytologists and many have approached the issue thoughtfully and prudently.

## 2. What is not highlighted and what this review would answer?

This review synthesizes the available legal cases and presents to practicing cytologists a short, relatively concise summary in the format of a hypothetical case. The review seeks to incorporate the legal issues with the practical aspects of running a cytology laboratory. The review answers how some state courts might approach the problem of lost and irreplaceable cytology slides, and offers general ideas to cytologist for minimizing the risk from lost slides.

## The big problem of the missing cytology slides

The issues surrounding the decision whether to send out diagnostic patient slides are more important in cytology than in surgical pathology because the cytology slides are typically unique and irreplaceable. Unlike surgical pathology cases, where additional paraffin sections can be cut, cytology slides often cannot be duplicated because there are only a few direct smears or the diagnostic material is present on a single slide.

Slides are routinely "sent out" for a variety of reasons. Most commonly, slides are sent to another institution because the patient's pathology slides will be reviewed before treatment. Some smaller laboratories with only one or two pathologist may send out slides as part of their quality assurance procedures. More rarely, but increasingly, slide's are requested as part of existing or contemplated litigation. Slides might be lost in any of these circumstances. A laboratory or hospital might loose the slides and not discover their absence until the slides are requested.

This article will discuss the approach the US legal system takes in addressing what happens when cytology slides are lost, and what steps a prudent laboratory might take to manage the risk. The article also intends to improve cytopathologists' awareness and understanding of some of the legal issues that arise when evidence is missing and perhaps promote an international comparative discussion of alternative legal approaches.

In the US, most of medical malpractice law is made and interpreted by state legislatures and state courts. What follows is a hypothetical story based on several legal decisions made by appellate state courts in the United States and reported in the legal literature. Although based on real cases and available to the public, the names have been changed. Importantly, none of this is intended as legal advice and readers should consult their attorney about specific questions.

### Facts

Rugged Labs (RL) is a small, independent laboratory. Part of Rugged Labs' work involves providing Big Giant Lab (BGL) with overflow services for cytopathology. In December of 1995, BGL bought out RL.

In 1994 and 1995, a RL cytotechnician interpreted two Pap smears from 30-year-old Ms. Penny as normal. In 1996 a cervical biopsy from Penny showed adenocarcinoma. Ms. Penny underwent a radical hysterectomy, which confirmed the invasive endocervical adenocarcinoma. Ms. Penny is alive today, but endured extended post-operative hospitalization.

At the time of the hysterectomy, Penny Plaintiff's oncologist requested that the 1994 and 1995 PAP smear slides be sent to Dr. Experta, who interpreted both Pap smears as containing "abnormal cell groups consistent with adenocarcinoma." Dr. Experta also reviewed the biopsy and in a note concluded that the cells on the Pap smears were consistent with the adenocarcinoma diagnosed on the cervical biopsy.

Approximately 6 months later, Plaintiff Penny decided to sue for medical malpractice based on failure to diagnose her endocervical adenocarcinoma on the Pap smears.

At some time before the plaintiff filed her lawsuit Dr. Experta's assistant apparently mailed the slides back to BGL. The Pap smear slides are lost, presumably in the mail, by BGL or Dr. Experta's office.

RL, the independent laboratory, asked the trial court to grant it summary judgment on the basis that the evidence was lost and no questions of fact remained. Summary judgment means that there are no outstanding questions of fact and the court needs to decide only questions of law. Summary judgment means there is no trial with a jury or judge hearing and weighing evidence. The question of law RL wanted the court to determine on summary judgment was that the lost slides substantially prejudiced RL and summary judgment was, therefore, appropriate. RL included in its motion for summary judgment an affidavit from an expert stating that she could not give an opinion without having the slides to look at. An affidavit by Dr. Experta's secretary stated that the slides were returned to BGL by US mail. BGL submitted an affidavit attesting they did not lose the slides. The parties to the lawsuit stipulated that the slides were lost. The trial court agreed with RL that there should be summary judgment in RL's favor, reasoning that no questions of fact needed to be answered and that a trial would unduly prejudice RL because of the spoliation of evidence. For a summary of the facts, please see Fig. 1.

### The Appellate Court's Opinion

The Plaintiff appealed and the state appellate court reversed the trial court, concluding that the trial court made a mistake in not allowing the case to go to trial. The appeals court reasoned that the case turned on a question of fact; "The slide either showed the presence of cancer cells or it did not." The appellate court envisioned the trial as follows: the cytotechnologist from RL would "testify to her conclusions" and the plaintiff would have Dr. Experta testify to her conclusions. There should be a trial because there were questions of fact including, in the words of the majority, whether the slide had "cancer cells" or not.

The majority also discounted the affidavit from BGL, stating that the conclusory statement that BGL had not lost the slides was not valid; just because BGL couldn't find the slides did not mean they never had them. In a footnote, the majority noted that although they aren't accusing anyone, they couldn't help but notice that missing the slides benefited BGL and RL.

One judge dissented in the three-judge panel that decided the case. The dissent focused on two points. First, the dissent emphasized that the defendant RL had nothing to do with loosing the slides. Although the plaintiff, Ms. Penny, had no "direct role" in the loss of evidence, the dissent treated Dr. Experta as an agent of the plaintiff and concluded that Dr Experta should have sent the slides back to RL not GBL. The dissent also reasoned that its approach would "encourage experts to treat more carefully evidence delivered into their hands."

Secondly, the dissent emphasized that the defendant RL is disadvantaged by the loss of the slide and the plaintiff has gained a significant advantage. The dissent sees a trial where the cytotechnologist's faces a serious credibility problem because her testimony will come across as blatantly self-serving. The cytotech will be limited to "opining that he made no error." Moreover, the fact-finder may conclude that the cytotechnologist is wrong since many may presume that the expert pathologist must be right. Finally, the defendants cannot obtain their own expert to bolster their case, because no cytology slides remain for review.

The dissent concludes that summary judgment for RL was proper because the trial court had good reasons to conclude that the lost evidence was going to unduly prejudice the defendant RL.

### Issues

The most obvious issue this case brings up is the difficulty in deciding what to do when it isn't clear who lost the evidence, or when a third party lost the evidence. In contrast, is the situation where one party is responsible for inadvertently losing the evidence or, even worse, where one party deliberately loses or destroys evidence. This is called spoliation of evidence and includes meaningful alteration of the evidence.

The court will determine the severity of the sanction for spoliation by the degree of willfulness or bad faith and the extent of the prejudice suffered by the non-responsible party. For example, in one case, surgeons performed a hepatectomy after a small needle core liver biopsy was interpreted as cancer. The hepatectomy specimen showed only cirrhosis and no cancer was found. The small needle liver biopsy was subsequently lost by the hospital. The patient sued for medical malpractice, claiming that the core biopsy was misdiagnosed and lead to an unnecessary hepatectomy. The trial court instructed the jurors that they could "draw the strongest possible inference against [the hospital] as to what the lost cytology slides would have shown." In other words, losing the slide means your opposition can make the slide show whatever they want. Interestingly, in the hepatectomy case, the defendants prevailed by arguing that the diagnosis of cancer and the decision to undergo resection were reasonable, regardless of the cytology results, because the patient had active hepatitis B and a suspicious liver mass on imaging. They argued that even with a negative cytology result the surgeons would have gone ahead with the hepatectomy.

But our case is different. Neither party, according to the majority, is responsible for losing the slides, or put another way, either party might be responsible for losing the slides. Several judicial options exist and none are neutral. One is to impose no sanctions and to proceed as usual, only without the slides, as the majority opinion advocated. This likely favors the plaintiff, particularly when there is a subsequent surgical specimen with cancer. A second option is to try to determine which party the court thinks is more prejudiced by the lost slides and then impose a legal remedy, as the trial court did in our hypothetical case by granting summary judgment. The difficulty is that it will not be clear which side is more prejudiced until the slide is recovered. The critical question of whether the negative diagnosis fell below the standard of care can likely be adequately answered only if the parties and their experts can review the slide. A third option might be to not allow Dr. Experta's testimony if the defendant can show that the expert knew or should have known that there was going to be litigation [1]. Arguably Dr. Experta should have returned the slides with greater care, regardless of whether litigation was contemplated or whether, as it seems in this case, the slides were sent to routinely review pathology slides before treatment.

Interestingly, each option leads to a different result based on bias about which party is ultimately more responsible or more likely responsible for loosing the slides and a bias about the standard of care. The majority's opinion included a note that the benefit to BGL from losing the slides can't be ignored, a clear statement that the plaintiff had no responsibility for losing the slides, and a simplistic view about the standard of care reflected by the statement that "the slide either showed the cancer cells or it did not." The majority's subtext is that the plaintiff did nothing wrong and they weren't completely sure about the laboratory. The dissenting opinion, in contrast, treated Dr. Experta as the plaintiff's agent and was dissatisfied that Dr. Experta returned the slide to BGL instead of RL, even though at the time BGL had purchased RL. The dissent also showed a more nuanced understanding of the standard of care, conveying skepticism about Dr. Experta's look back conclusion that the Pap smears were "consistent" with adenocarcinoma. The dissent reasoned that perhaps the cells are consistent with malignancy only in the retroscope and that a defense expert might reasonably conclude that it was "not below the standard of care to determine the biopsy negative" were the slides available for review.

### Comment

The facts often surrounding a cytopathology medical malpractice case are that there is a subsequent biopsy or surgical specimen with a discrepant diagnosis. If, as in the hypothetical *Penny vs. GBL*, a court does not dismiss the plaintiff's case, the absence of the cytology slide will likely impact the defendant cytologist more adversely because many people will assume that the cancerous cells were on the slide and the cytologist or cytotechnologist missed the cancer, which, after all, was present on the subsequent biopsy. In the hypothetical's facts, this was particularly true since Dr. Experta had already opined that the Paps were "consistent with adenocarcinoma." The defendant cytologist/cytology lab is at a serious disadvantage if the court allows Dr. Experta's opinion as admissible evidence. The majority's comment that GBL benefited from the lost slides and the appellate court's decision to allow the plaintiff to go to trial, suggests the court's bias that they believed Dr Experta's interpretation was the correct one.

Admittedly, a cytology lab or cytologist does benefit if slides are lost and the court does not attach any responsibility for losing the slides to the potential laboratory or cytologist defendant, because the plaintiff will not have enough evidence to prevail. Similarly, if the slides are discarded after the legal time periods the likelihood of a successful lawsuit is slim because the plaintiff will not have enough evidence, and the defendant complied with legal requirements regarding slide retention. The hypothetical case of Penny vs GBL differs. Remember that once the court allows the case to go to trial, GBL only benefits from absent slides if the defendant initially misinterpreted the slides. If the slides contained no malignant cells, and Dr. Experta over-interpreted the slides, then GBL is prejudiced by not being able to show the slide.

Cytology slides are typically in possession the cytology labs. The slides may be sent out for a variety of reasons, but at least initially the lab has possession of the cytology slides. This is both an advantage and a disadvantage for the lab. The disadvantage is that if slides are lost while in the lab's possession a court will typically see it as the lab's responsibility to safeguard the slides. This allows the plaintiff to have the jury infer whatever is best for the plaintiff's case; that the slide had malignant cells when the cytology diagnosis was benign or that there were only benign cells when the diagnosis was malignant, as in the hepatectomy case.

The advantage for the cytology lab is that it is in a position of relative control. The lab can implement a system to help reduce the chances of losing a slide and reduce the risk if a slide is lost. Although the lab may want to employ the help of an attorney experienced in these matters, there are several steps every cytology lab can take. First, the lab should ensure that cytology slides are retained for the time that the current federal CLIA regulations, applicable state regulations and the CAP checklist require. All glass cytology slides must be retained for at least 5 years and fine needle aspiration slides retained for 10 years. (Some state regulations may require longer times.) The CAP checklist also requires policies for "protecting and preserving the integrity and retrieval of original slides in cytopathology" and "to ensure defined handling and documentation of the use, circulation, referral, transfer and receipt of original slides to ensure availability of materials for consultation and legal proceedings." Keeping careful records about when and which slides are released to whom is essential for reducing the risk of losing slides. In the send out cases where slides are sent out for a routine second opinion not sought in contemplation of a law suit or because a patient will be treated elsewhere, the lab might obtain the borrower's explicit written agreement that the borrower has responsibility for the slides with an explicit provision about a duty to indemnify the cytology lab for any losses due to a lost slide or slides. A documented telephone call to request the return of tardy slides may also be worthwhile. In cases where slides are requested in contemplation of a lawsuit, the lab may be able to implement a policy that review of slides is done at the lab, ensuring that the lab retains possession. Alternatively, the lab may pursue or agree to a court order requiring production of the slides that clearly addresses who is responsible for the slides and includes an indemnity clause in the event the slides are lost.

It is reasonable to make a distinction between sending out non-reproducible slides to an institution that will treat the patient and sending out non-reproducible slides to the plaintiff's expert witness. It is, therefore, important that the lab understands the purpose for which the slides are requested. A documented telephone conversation may clarify the purpose of the outside review and allow the lab to appropriately "triage" the case.

Laboratory administrators should understand that the cytology lab serves a public function in safeguarding cytology slides. Court's have recognized a public policy reason to have the laboratory safeguard slides, in part so that the slides are available in the event of malpractice litigation. At the same time, many states have statutes that give patients the right to examine and copy their medical records (the recent federal HIPAA does the same). These two propositions are not mutually exclusive. One state case considered the question of whether a patient/plaintiff had a right to "immediate possession of pathology slides" and concluded that the plaintiff did not. The court decided that the patient's rights did not exceed the patient's statutory right in the slides. In other words the court was not going to find a common law, or customary, right in the slides that gave the patient a greater right than the applicable statute. The decision noted that the legislative history of the statute included remarks that pathology slides were part of the medical record. The judges then approached the second question about what to do when the "medical record" cannot be duplicated, as with a Pap smear or other cytology slide. In answering this question, the court noted that hospitals and laboratories have public as well as private duties. One of their public duties is to retain slides so that the slides are available in the event of malpractice litigation. This meant that patients do not have a legal right to possess parts of the medical record that cannot be duplicated. The court, however, did not grant the lab complete authority to never release the slides. Since the public policy reason depended fundamentally on preserving slides to help the legal system run smoothly, the decision reminded the laboratory or hospital that, pursuant to the clear terms of a statute, it must send the original slides to a "licensed institution, laboratory or physician" at the patient's written request.

The dissent characterized the pivotal issue differently and concluded that the patient had a right to immediate possession because the slides contained the patient's cells and she had the right "to control one's body." The dissent reasoned that recent advancement in genetic science and the accompanying difficult privacy issues raised by genetic information strengthened the patient's right to possession of the cells on the glass slide. The majority addressed this argument and, citing the well known case of *Moore vs Regents of the University of California *[2], reminded the reader that no court has recognized that a patient has property rights in cells taken for diagnostic purposes. I mention the dissent to emphasize that the issues surrounding possession and use of glass slides are complex and evolving and it often difficult to predict what a court will say.

In summary, lost slides can be a problem for cytology laboratories and cytologists, whether responsible for losing the slides or not. The prudent cytologist will minimize the risk of lost slides, because, as the hypothetical case of *Penny v GBL *illustrates, lost slides can result in problems for cytologists and the cytopathology laboratory A good place to start is to ensure that existing CLIA regulations, applicable state regulations and other guidelines, such as the CAP checklist, are in place in the laboratory. Thinking about the issue and implementing appropriate risk management strategies with the help of an attorney are also prudent measures.

**Table 1:** The top 10 take home messages

1. Spoliation of evidence includes meaningful alteration of the evidence as well as losing the evidence.
2. A court may look at which party benefits from losing the unique and irreplaceable slides as well as which party was last in possession of the slides and impose appropriate sanctions.
3. One remedy a court may impose on the party responsible for losing the slides is to allow the factfinder, whether jury or judge, to infer that the slides show whatever is best for the opposition's case.
4. Cytologists should adhere to federal, state and respected published checklists regarding how long to keep slides and implement lab policies regarding the circulation and transfer of original slides.
5. A cytology lab should consider calling to find out the reason why a slide is requested so that it can respond appropriately.
6. A cytology laboratory should consider sending unique and irreplaceable cytology slides by registered mail.
7. Some state legislatures and courts recognize a public policy reason for cytology labs to retain and protect cytology slides.
8. Although patients have a right to inspect slides, they typically do not have a legal right to immediate possession.
9. A cytology lab should consider implementing a policy that requires review of the slides at the lab when slide review is in contemplation of a law suit. If this is not possible, then the lab can agree to a court order that clearly spells out who is responsible if slides are lost with indemnification to the lab for lost slides.
10. When in doubt, the prudent cytologist should contact their attorney.
